# Editorial: Biomolecules Against Coronaviruses: Molecular Aspects, Multi-Omics and Systems Pharmacology

**DOI:** 10.3389/fphar.2021.835884

**Published:** 2022-01-12

**Authors:** Christophe Hano, Ilaria Peluso, Jen-Tsung Chen

**Affiliations:** ^1^ Collegium Sciences et Techniques, Université d’Orléans, Eure et Loir Campus, Chartres, France; ^2^ Research Centre for Food and Nutrition, Council for Agricultural Research and Economics (CREA-AN), Rome, Italy; ^3^ Department of Life Sciences, National University of Kaohsiung, Kaohsiung, Taiwan

**Keywords:** coronaviruses, bioactive compounds, multi-omics, bioinformatics, natural products, systems pharmacology, traditional medicines

## Introduction

Viruses are widespread small infectious particles ranging in size from 20 to 300 nm, containing nucleic acids, proteins, and lipids. Despite of their quite simple structure, their interactions with their host are highly sophisticated. Viruses represent a significant danger to the economy and global health because of their tendency for epidemics and pandemics, as well as their propensity for mutation and vaccination and medication resistance. Over the past two decades, several viruses have been reported to have triggered epidemics or pandemics, including for examples those caused by avian Influenza A (H5N1) in 1997, paramyxovirus (Nipah virus) in 1999, coronavirus (CoV), also known as SARS-CoV, in 2002, swine H1N1 Influenza A virus in 2009, Middle East respiratory syndrome virus (MERS-CoV) in 2012, Ebola crisis in 2014 (see Thomas et al., 2021 for review). The last is the current SARS-CoV-2, also known as COVID-19.

CoV is the umbrella term for a collection of RNA viruses that can cause anything from a simple cold to a life-threatening illness. COVID-19 has turned into a global public health crisis. It has infected more over 260 million individuals throughout the world, resulting in five million deaths as of December 2021 (World Health Organization, 2021a). There are already some vaccination options, however the targeting of few viral proteins has significant downsides due to the rapid mutations of the virus. Therefore, new therapeutic drugs are urgently needed, and a SARS-CoV-2 antiviral drug that has been clinically validated is still on the way. Identifying active biomolecules may be accomplished through a variety of approaches. This was the focus of the present research topic on Biomolecules Against Coronaviruses: Molecular Aspects, Multi-omics, and Systems Pharmacology.

## The Drugs Repurposing Approach

Drug repurposing is an option consisting in looking at the possibility of repurposing existing antiviral drugs, that can help speed up the drug development process and save time and money. However, only a few antiviral medications are available, such as acyclovir for herpes simplex virus treatment or amantadine for influenza type A treatment. None of these are effective against all types of viruses. As a result, discovering new antiviral medications is critical. Thus, several drugs (e.g., oseltamivir, ritonavir, remdesivir, ribavirin, favipiravir, chloroquine, hydroxychloroquine) have been proposed and evaluated for the treatment of COVID-19. In the present research topic, Khan et al. proposed that one of these drugs, remdesivir, strongly binds to membrane protein (Mprotein), RNA-dependent RNA polymerase (RDRP), and main protease (Mprotease) of SARS-CoV-2. I It is crucial to remember, however, that the World Health Organization (2021b) recommends against the use of remdesivir.

## Nature and Traditional Medicines as Reservoirs of Active Biomolecules

Exploring natural compounds may be an effective strategy to discover novel antiviral drugs. Natural products have been utilized for therapeutic reasons across the world for a long time. Plant compounds are the most significant natural sources for the discovery of novel therapeutic compounds due to their chemical and structural diversity. Morphine, quinine, paclitaxel, vinblastine, penicillin, digitoxin, lovastatin, berberine, and doxorubicin are only a few examples of natural compounds discovered as lead drugs. Furthermore, several natural chemicals have been employed as scaffolds for the synthesis of novel synthetic medications, including chloroquine, atorvastatin, etoposide, aptopril, aspirin, and pentazocine (Newman and Cragg, 2007).

Chen et al., in their review, nicely illustrated the interest of bioactive molecules of natural origin, particularly medicinal plants, as potential resources in the treatment of SARS-CoV-2, acting at different stages of the viral life cycle and targeting various viral or host targets, offering a promising approach to drug development. In addition to basic biological information on SARS-CoV-2, such as the virus biological properties and methods of invasion, the authors properly reviewed previously reported natural bioactive compounds with potential anti-coronavirus activities, organizing them according to their various targets in the viral infection life cycle of human cells, and examining their potential to treat COVID-19.

Among the plethora of active phytochemicals, Zheng et al. illustrated that glycyrrhizic acid (GA) may be effective against SARS-CoV-2 due to its anti-inflammatory and antiviral properties, as well as its ability to alter critical host interaction proteins. However, the method by which GA controls host factors has yet to be uncovered. According to the pathway enrichment analysis results, GA’s antioxidant, antiviral, and anti-inflammatory properties, as well as its capacity to boost the immune system, may help to avoid cytokine storms induced by COVID-19 infection.

Nallusamy et al. used a virtual screening methodology to examine at the antiviral potential of 605 phytochemicals from 37 plant species and 139 compounds to inhibit several SARS-CoV-2 protein targets. The authors proposed a phytochemicals shortlist that might be used as a starting point for future therapeutic research and development. The broad-spectrum inhibitory activity of certain phytochemicals, such as cyanine, on the major proteases of several coronaviruses was investigated, whereas phytochemical sources as potential multi-target inhibitors against SARS-CoV-2 are mentioned. This research also revealed that the traditional herbal mixture “Kabasura kudineer” may offer antiviral properties. This illustrates the widespread use of plants in traditional medicine to treat, diagnose, and prevent disease, as well as to preserve health.

Traditional medicines, as proven by the success of the antimalarial artemisinin and the Nobel Prize for Prof. Tu Youyou’s work, are a vital source of inspiration for so-called contemporary medicine, which can greatly contribute to the (re)discovery of novel medications (Tu, 2015). It is important to realize that artemisinin is not an isolated case. Many plants have been employed as essential components in traditional Chinese, Indian, Japanese, Thai, Korean, African, Native American, or European medicines, and many unknown bioactive compounds have been identified as a result of this traditional knowledge (Buenz et al., 2018). Several attempts have been made over the last 2 years to evaluate traditional herbal remedies known for their health benefits and immune-boosting action against SARS-CoV-2, including testing traditional herbal remedies known for their antiviral and/or anti-inflammatory properties, health benefits, and immune-stimulating activity.

Hu et al. reported a clinical study that found a mix of Western and traditional Chinese remedies to be beneficial in treating SARS-CoV-2, including the effects of nine herbs in the He-Jie-Shen-Shi (HJSS) decoction used to treat severe COVID-19. From February to March 2020, 81 patients with severe COVID-19 were recruited for retrospective cohort study at Wuhan Tongji Hospital with the aim to determine the clinical effectiveness of HJSS combination therapy with Western monotherapy in the treatment of severe COVID-19, as well as to look into HJSS’s likely mechanism of action. Several putative targets were identified and may be involved in therapeutic pathways including viral replication, inflammatory response, and oxidative stress, as well as lung damage reduction. Moreover, the presence of *Glycyrrhiza uralensis* Fisch. ex DC. [Fabaceae], the main source of GA, which has already been reported as a potential anti-viral against COVID-19 by Zheng et al., among the plants studied in this work is noteworthy. More research is needed to confirm the therapeutic value and mechanisms of action, but this study nicely illustrates the contribution of medicinal plants used by Traditional medicines.

## The Contribution of Omics Approaches to Discover New Biomolecules and Their Actions

Singh et al. described how omics/multi-omics approaches may be utilized to investigate the therapeutic potential of biomolecules in the treatment and prevention of SARS-CoV-2 infection. Efforts have been done in the last 2 years to identify new therapeutic substances by repurposing existing antiviral drugs and/or identifying new leads derived from plants. During these efforts, SARS-CoV-2 whole-genome sequencing provided a path for exploring omics systems and strategies to solve this global health emergency. Investigations of genome, proteome, and metagenome sequences have contributed in the identification of viral nature, which has aided in the understanding of the disease’s molecular mechanism, structure, and dissemination. Multi-omics approaches may be used to uncover potential COVID-19 medicinal biomolecules. Moreover, herbal extracts are complex, including a mixture of active biomolecules. Most of the time, extract effect is due to the synergistic combination of different bioactive components, rather than just one. In viral epidemics or pandemics, omics approaches may be used to ease the discovery therapeutically active plant biomolecules. The ideas and uses of omics techniques for discovering therapeutically active plant biomolecules in infectious epidemics or pandemics are outlined by the authors of this review.

## Futures Directions

The coronavirus outbreak has raised several issues that naturally causes anxiety, but it also inspires optimism. The great majority of emerging diseases that have given rise epidemics are zoonoses, or infections that are transmitted by animals. This increase in the frequency of epidemics may be attributable in part to human activities that alter the environment and enhance the risk of a human-pathogen interaction. Global warming may also be a concern in some circumstances since it is linked to a future pandemic risk, as it has the ability to speed up the migrations of wild animals that serve as potential virus reservoirs, as well as population movements, boosting disease dissemination. However, it is also an opportunity to reconnect with our environment and (re)discover what it can offer. No one can anticipate what will happen next or when the pandemic will stop, but our history has proven that natural biomolecules are an almost infinite source of inspiration for the development of new and successful drugs and therapeutic strategies. Only around 15% of natural sources have been investigated so far, giving ample opportunity for further research. A plethora of data on the identification of biomolecules from natural sources with antiviral properties has been released in the last 2 years. We assume that natural herbal biomolecules, with the support of advanced methodologies such as multi-omics and/or virtual screening approaches, might play a key role in the creation of new effective antiviral drugs.
